# Effects of dietary supplementation with fermented and non-fermented brown algae by-products on laying performance, egg quality, and blood profile in laying hens

**DOI:** 10.5713/ajas.17.0921

**Published:** 2018-04-12

**Authors:** Yongjun Choi, Eun Chae Lee, Youngjun Na, Sang Rak Lee

**Affiliations:** 1 Department of Animal Science and Technology, Konkuk University, Seoul 05029, Korea

**Keywords:** Seaweed, Fermentation, Egg Production, Haugh Unit, Laying Hen

## Abstract

**Objective:**

This study was conducted to investigate the effects of dietary supplementation with fermented and non-fermented brown algae by-products on the laying performance, egg quality, relative organ weight, and blood profile of laying hens.

**Methods:**

Hy-Line Brown chickens (n = 180; 70-week-old) were randomly divided into 5 groups with 4 replicates per group (3 hens per cage, 4 cages per replicate), and fed with 5 experimental diets, namely the basal control diet (CON) or the control diet supplemented with 0.5% brown seaweed (BS), 0.5% seaweed fusiforme (SF), 0.5% fermented brown seaweed (FBS), or 0.5% fermented seaweed fusiforme (FSF), for 4 weeks.

**Results:**

Egg production rate and egg mass were greater in the BS group than in the other groups (p<0.05), and the SF and FSF groups had greater egg production than the control group (p<0.05). Egg weight was higher in the BS group than in the other groups (p<0.05). There were no differences in eggshell color, egg yolk color, eggshell strength, or eggshell thickness among the groups. There was no difference in Haugh units among the treatment groups, except for the FSF group, which had a significantly lower value (p<0.05). The non-fermented groups had greater relative organ weights, particularly the liver and cecum, than the other groups (p<0.05). Regarding blood profile, the supplemented-diet groups had higher albumin levels than the control group (p<0.05). The FBS group had higher total cholesterol and triglyceride levels than the other groups (p<0.05). The BS and FBS groups had higher glutamic pyruvic transaminase levels than the other groups (p<0.05).

**Conclusion:**

This study demonstrated that dietary brown algae supplementation can improve egg-laying performance; however, supplementation with fermented seaweeds had no positive effect on the egg-laying performance of hens.

## INTRODUCTION

Feed additives and antibiotics have been used to improve growth performance and enhance animal health in the poultry industry. As consumer demand for food safety has increased, it has caused to change the way of livestock husbandry systems. The use of antibiotics is recognized as a problem in the livestock industry. As the use of antibiotics has been banned or decreased, many studies have been conducted with the aim of increasing the immunity and productivity of animals by feeding with supplements or additives [1,2].

Brown algae are mainly produced and consumed in Asia, and a large quantity of by-products (e.g., roots and stems) are created during the production process. The majority of brown seaweed (BS) by-products in domestic markets are disposed of; more than 60,000 and 10,000 tons per year of *Undaria pinnatifida* and *Hizikia fusiforme* by-product respectively are produced in Korea. The BS (*Undaria pinnatifida*) and seaweed fusiforme (SF; *Hizikia fusiforme*) are representative brown algae, and they are known to have benefits for animals. Most brown algae, including BS and SF, contain various minerals and organic acids (e.g., alginate and fucoidan). Alginate, which is enzymatically converted to alginate oligomers, has a stimulatory effect on cytokine secretion in immune cells [3], and it has a positive effect on the immune system through the improvement of zinc bioavailability [4]. Fucoidan, present in BS, has been reported to help in blood coagulation [5], and has both anticancer [6] and antioxidant effects [7]. Furthermore, a previous study reported an improvement in meat quality in broiler chickens [8]. For these reasons, brown algae are considered to have potential as animal feed additives.

Fermentation is used to increase the storage period of feed using lactic acid bacteria. Fermentation has been reported to not only increase feed storage period, but also to enhance the antioxidant, anticoagulant [9], and anti-inflammatory [10] effects of brown algae. Furthermore, the effects of probiotics using microbial fermentation can have a positive effect on poultry performance [11]. Therefore, fermentation of brown algae using various microorganisms might be expected to have a synergistic effect as a feed additive for poultry.

Studies on the effects of fermented brown algae are extremely rare in livestock. Therefore, this study was conducted to investigate the effect of dietary supplementation with fermented and non-fermented brown algae (*Undaria pinnatifida* and *Hizikia fusiforme*) by-products on the productivity of laying hens.

## MATERIALS AND METHODS

### Seaweeds and fermentation

By-products of BS and SF were collected from Wando island (South Korea). Fermentation of BS was performed using *Bacillus subtilis* at 37°C for 48 h and anaerobic stress. Fermentation of SF was performed using *Aspergillus oryzae* at 37°C for 96 h and aerobic stress. The chemical composition of the supplement is shown in Table 1. Seaweed decomposition was calculated from the ratio of reduced sugar. The end-point of fermentation was determined from total sugar, reduced sugar, and pH according to the method of Choi et al [12]. Fermentation conditions were handled according to the optimum growth conditions from the American Type Culture Collection (ATCC) guidelines [13]. A light microscope (Olympus, Nishi-Shinjuku, Japan), and scanning electron microscope (Phillips, Andover, MA, USA) were employed to compare seaweed by-products after fermentation. The fermented seaweed (*Undaria pinnatifida* and *Hizikia fusiformis*) by-products were stored at −20°C until supplementation.

### Animals, diet, and management

A total of one-hundred and eighty 70-week-old Hy-Line Brown layers were divided into 5 groups and fed with the control diet or experimental diets (non-medicated diet containing 0.5% seaweed). The experiment was performed using a completely randomized design with a control and 2×2 factorial arrangement. Each treatment included 4 replicates with 3 cages each and 3 hens per cage (width×length = 47×40 cm; 626.6 cm^2^/hen) such that each treatment involved 36 hens (9 hens/replicate, 36 hens/treatment). The treatments included the basal control diet (CON), control+0.5% BS by-product, control+0.5% FBS by-product, control+0.5% SF by-product, and control+ 0.5% fermented seaweed fusiforme by-product (FSF). The experimental diets were formulated according to the National Research Council guidelines [14] (Table 2). Food and water were provided *ad libitum*. A room temperature of 21°C±3°C and a photoperiod of 16/8 h light/dark cycle were maintained throughout the experimental period. Experimental diets were provided daily, and feed intake was recorded each week. The experiments were performed in compliance with the guidelines of the Institutional Animal Care and Use Committee of Konkuk University (KU15186).

### Egg production and quality

The number and weight of eggs were recorded daily, and abnormal eggs were excluded from egg weight measurements. Egg mass was calculated as the hen-day egg production multiplied by the average egg weight. Five eggs were collected from each replicate every two weeks, weighed individually, and stored at room temperature before subsequent measurements.

The eggshell strength of intact eggs was measured using an eggshell strength tester (FHK, Fujihara Ltd., Tokyo, Japan). Eggshell thickness without the shell membrane was measured in micrometers using eggshell fragments, after measuring eggshell strength (FHK Peacock, Fujihara Ltd., Japan). Albumen height and eggshell color were evaluated using an Egg Multi Tester (QCM+ Technical Services and Supplies Ltd., York, England). The Haugh unit, along with albumen height per egg weight value, were calculated using the method of An et al [15]. Egg yolk color was determined using a Roche yolk color fan (Hoffmann-La Roche Ltd., Basel, Switzerland).

### Sampling and measurements

Two birds per replicate (eight birds per treatment) were selected and weighed individually. The liver, spleen, cecum, and abdominal fat were evaluated using samples obtained from these chickens at the end of the experiment. Organ weight was expressed as g/100 g body weight. Blood samples were collected from the brachial vein in 5-mL vacutainer tubes. Serum was obtained through centrifugation at 2,000×*g* for 15 min and stored at −70°C until analysis. Serum was analyzed to compare the levels of albumin, globulin, total cholesterol, glutamic oxaloacetic transaminase (GOT), glutamic-pyruvic transaminase (GPT), and blood urea nitrogen (BUN) using an automated blood analyzer (Sysmex, Seoul, Korea).

### Statistical analysis

Data were analyzed using the MIXED procedure of the SAS package [16] in a completely randomized design. The model used was

$$\text{Y}=\mu +{\text{T}}_{\text{i}}+{\text{E}}_{\text{ij}}$$

Where μ is the average value, T_i_ is the treatment value, and E_ij_ is the error value. The fixed effect was the supplement effect; a random effect was not considered in the procedure. A multiple comparison test was used to compare between each treatment using the PDIFF option. Significant differences among the treatments were determined at p<0.05, whereas a trend was observed when 0.05<p<0.10. All means presented are least squares means.

## RESULTS AND DISCUSSION

### Laying performance

There was no significant difference in feed intake among the treatment groups. The BS group had higher egg production than the other groups (p<0.05), and both the SF and FSF groups had higher egg production than the control group (p< 0.05) (Table 3). Egg weight and egg mass were higher in the BS and SF groups than in the control group (p<0.05); fermentation had a negative effect on egg weight in laying hens (p< 0.05). Egg performance did not appear to be affected by feed intake as there was no difference among treatments. Brown algae is reported to contain a rich polysaccharide. Complex polysaccharides in the feed are resistant to acid hydrolysis and can result in gastrointestinal influx [17]. The oligosaccharide-rich in gastrointestinal tract improved immune status, growth performance and gut microbiota [18]. For these reason, in this study, it was considered that oligosaccharide-rich have a positive effect on laying performance. The mode of action of probiotics was known that includes maintaining intestinal microflora by antagonism, changing metabolism by increasing enzyme activity and decreasing ammonia production and bacterial enzyme activity, enhancing feed intake and digestion, and neutralizing enterotoxins and stimulating the immune system [19]. Although fermentation as probiotics was reported many positive effects in poultry, the result of this study was shown that fermentation have a negative effect on laying performance compare with control and non-fermented group. In this study, fermentation using bacteria was caused consuming oligosaccharides, it seems to be decreased supplementation effect as prebiotics [20].

### Egg quality and relative organ weight

There was no significant difference in eggshell color, egg yolk color, eggshell strength, and eggshell thickness among the treatment groups (Table 4). Eggshell formation was reported to be affected by minerals, such as calcium, phosphorus, magnesium, potassium, and so on [14]. In this study, dietary supplementation with BS and FS included many minerals necessary for eggshell formation [21]. However, in this study, it was considered that there was no difference in egg quality due to all minerals needed for egg shell formation were supplied in all experimental feeds.

The relative weights of the cecum the non-fermented diet supplemented groups showed greater the relative cecum weights than the other treatment groups (p<0.05). There was no significant difference in relative liver, spleen and abdominal fat weight among the treatments groups (Table 5). In previous study, As the level of the oligosaccharide increased in the experimental feed, the weight of the liver increased in broiler chicken [22]. the increase in relative liver weight might be explained by the synthesis fat content [23]. However, in this study, there was no consistent association between liver weight and abdominal fat. the increase in relative cecum weight might be explained by difference of oligosaccharides magnitude among all treatments, the oligosaccharide-rich in cecum improved immune status, growth performance and gut microbiota [17]. It was considered that increasing cecum influx polysaccharide have effect on increasing microbial population.

### Blood profiles

The BS groups showed the greatest blood albumin levels among all treatment groups (p<0.05) and the diet supplemented groups had greater than control group (p<0.05) (Table 6). The FBS groups showed the greatest total cholesterol and triglyceride among all treatment groups (p<0.05) and the BS and FSF supplemented groups had greater than control group (p<0.05). There was no significant difference in globulin, aspartate aminotransferase (AST/GOT), and BUN levels among the treatment groups. The BS and FBS groups showed greater blood alanine aminotransferase (ALT/GPT) levels than the other groups (p<0.05). In previous study, as increasing BS level in feed was shown blood albumin level was increased in laying hen [24] and similar results were obtained in this experiment. Although, however, albumin level of supplementation groups was higher than control, it included the normal range of albumin level in laying hen [25]. In previous study, BS was reported that reduced AST (GOT) and ALT (GPT) in blood serum [26]. However, ALT (GPT) of BS treatment group was shown that was higher than other groups. The difference of ALT (GPT) might be explained by the synthesis fat content (Table 5).

## CONCLUSION

Dietary supplementation with 0.5% seaweeds is concluded to improve egg production without affecting eggshell characteristics in egg-laying hens. Supplementation of fermented seaweeds had no positive effect on the laying performance in laying hens.

## Figures and Tables

**Table 1:** The chemical composition in the by-products of brown seaweed, seaweed fusiforme, fermented brown seaweed and fermented seaweed fusiforme

Chemical composition (% DM)	Treatment			

	BS	FBS	SF	FSF
Crude protein	17.38	18.41	18.41	20.04
Crude fat	0.57	0.85	0.11	0.12
Carbohydrate1)	29.84	45.13	40.73	49.20
Crude ash	28.42	20.22	16.63	15.04
Ca	0.93	1.27	0.87	0.93
P	0.28	0.37	0.11	0.12
Fe	0.59	0.98	1.43	0.16
Zn	0.24	0.31	0.15	0.17
Mg	0.69	0.01	0.01	0.01
K	0.63	0.41	0.32	0.36
Na	0.72	0.55	0.16	0.22

DM, dry matter; BS, brown seaweed by-product; FBS, fermented brown seaweed by-product; SF, seaweed fusiforme by-product; FSF, fermented seaweed fusiforme by-product.

1) Calculated value: [sample 100 g–(moisture+crude protein+crude fat+crude ash)]/100 g×100.

**Table 2:** Formula and calculated nutritional values of the basal diet

Items	Treatment1)				

	CON	BS	FBS	SF	FSF
Ingredients (%)					
Yellow corn	-	-	56.2	-	-
Wheat	-	-	7.6	-	-
Lupin seed	-	-	4	-	-
Feather meal	-	-	2.3	-	-
Soybean meal	-	-	14.5	-	-
Rape seed meal	-	-	3	-	-
Tallow	-	-	1.3	-	-
Calcium carbonate	-	-	9.95	-	-
Salt	-	-	0.25	-	-
Vitamin-mineral mixture2)	-	-	0.71	-	-
DL-methionine (98%)	-	-	0.14	-	-
Choline Cl-50 (50%)	-	-	0.05	-	-
Total	-	-	100	-	-
Calculated nutrients contents					
Moisture (%)	-	-	10.87	-	-
CP (% DM)	-	-	16.36	-	-
Ether extract (% DM)	-	-	4.23	-	-
Crude fiber (% DM)	-	-	3.22	-	-
Crude ash (% DM)	-	-	13.15	-	-
Ca (% DM)	-	-	4.09	-	-
P (% DM)	-	-	0.41	-	-
TMEn (kcal/kg)	-	-	2,753.00	-	-
Supplement					
MB (% DM)	-	0.5	-	-	-
FMB (% DM)	-	-	0.5	-	-
FB (% DM)	-	-	-	0.5	-
FFB (% DM)	-	-	-	-	0.5

CP, crude protein; DM, dry matter; TMEn, nitrogen-corrected apparent metabolizable energy.

1) CON, control (basal diet); BS, brown seaweed by-product; FBS, fermented brown seaweed by-product; SF, seaweed fusiforme by-product; FSF, fermented seaweed fusiforme by-product

2) The vitamin and mineral mixture provided the following nutrients per kilogram of diet: vitamin A, 12,000 IU; vitamin D_3_, 2,500 IU; vitamin E, 20 IU; vitamin K_3_, 1.8 mg; vitamin B_1_, 2.0 mg; vitamin B_2_, 6.0 mg; vitamin B_6_, 3.0 mg; vitamin B_12_, 0.020 mg; niacin, 25 mg; pantothenic acid, 10 mg; folic acid, 1.0 mg; biotin, 50 mg; Fe, 50 mg; Zn, 65 mg; Mn, 65 mg; Co, 0.250 mg.

**Table 3:** Effects of dietary supplementation with non-fermented or fermented by-products of brown seaweed and seaweed fusiforme on laying performance in laying hens

Item	Treatments					SEM	p-value

	CON	BS	FBS	SF	FSF		
Feed intake (g/d)	131.9	130.6	131.4	133.7	137.7	4.06	0.755
Egg production rate (%)	65.17^b^	73.83^a^	64.18^b^	68.21^ab^	66.75^ab^	1.88	0.004
Egg weight (g)	64.13^bc^	65.40^a^	63.63^c^	65.00^ab^	63.91^bc^	0.31	<0.001
Egg mass (g/d)	41.82^b^	47.94^a^	40.04^b^	43.16^ab^	41.88^b^	1.31	0.001

CON, control (basal diet); BS, brown seaweed by-product; FBS, fermented brown seaweed by-product; SF, seaweed fusiforme by-product; FSF, fermented seaweed fusiforme by-product; SEM, standard error of the mean.

abc Means followed by different letters in superscript in the same row differ significantly (p<0.05).

**Table 4:** Effects of dietary supplementation with non-fermented or fermented by-products of brown seaweed and seaweed fusiforme on egg and eggshell characteristics in laying hens

Item	Treatments					SEM	p-value

	CON	BS	FBS	SF	FSF		
Egg shell color (unit)	28.9	29.4	29.4	29.3	29.9	0.62	0.8823
Egg yolk color (RCF)	7.2	7.1	7.1	7.1	7.1	0.06	0.5934
Egg shell strength (kg/cm^2^)	2.2	2.1	2.1	2.1	2.1	0.08	0.6529
Egg shell thickness (mm)	0.4	0.3	0.4	0.4	0.3	0.00	0.4743
Haugh unit	80.8^a^	80.4^a^	80.4^a^	79.3^a^	76.4^b^	0.97	0.008

CON, control (basal diet); BS, brown seaweed by-product; FBS, fermented brown seaweed by-product; SF, seaweed fusiforme by-product; FSF, fermented seaweed fusiforme by-product; SEM, standard error of the mean.

ab Means followed by different letters in superscript in the same row differ significantly (p<0.05).

**Table 5:** Effects of dietary supplementation with non-fermented or fermented by-products of brown seaweed and seaweed fusiforme on relative organ weights in laying hens

Item	Treatments					SEM	p-value

	CON	BS	FBS	SF	FSF		
Liver (g/100 g BW)	2.10	2.45	2.14	2.42	1.94	0.16	0.119
Spleen (g/100 g BW)	0.10	0.09	0.06	0.09	0.10	0.01	0.168
Cecum (g/100 g BW)	0.71^b^	0.85^a^	0.70^b^	0.88^a^	0.76^ab^	0.05	0.020
Abdominal fat (g/100 g BW)	3.24	3.91	5.07	3.97	4.05	0.45	0.088

CON, control (basal diet); BS, brown seaweed by-product; FBS, fermented brown seaweed by-product; SF, seaweed fusiforme by-product; FSF, fermented seaweed fusiforme by-product; SEM, standard error of the mean; BW, body weight.

ab Means followed by different letters in superscript in the same row differ significantly (p<0.05).

**Table 6:** Effects of dietary supplementation with non-fermented or fermented by-products of brown seaweed and seaweed fusiforme on blood profiles in laying hens

Item	Treatments					SEM	p-value

	CON	BS	FBS	SF	FSF		
Albumin (g/dL)	2.02^c^	2.36^a^	2.28^ab^	2.25^ab^	2.17^bc^	0.06	0.002
Globulin (g/dL)	3.73	3.65	3.49	3.79	3.76	0.18	0.781
Total cholesterol (mg/dL)	125.1^c^	145.8^b^	198.2^a^	123.0^c^	168.5^ab^	11.6	<0.001
Triglyceride (mg/dL)	822.5^c^	1,542.0^b^	2,039.0^a^	835.0^c^	1,195.3^bc^	144.8	<0.001
AST (GOT) (IU/L)	184.5	163.5	202.7	175.3	166.8	11.9	0.153
ALT (GPT) (IU/L)	4.75^b^	11.00^a^	15.50^a^	4.14^b^	4.00^b^	1.77	<0.001
BUN	0.50	0.50	0.50	0.58	0.79	0.12	0.413

CON, control (basal diet); BS, brown seaweed by-product; FBS, fermented brown seaweed by-product; SF, seaweed fusiforme by-product; FSF, fermented seaweed fusiforme by-product; SEM, standard error of the mean; AST, aspartate aminotransferase; GOT, glutamic oxaloacetic transaminase; ALT, alanine aminotransferase; GPT, glutamic-pyruvic transaminase; BUN, blood urea nitrogen.

abc Means followed by different letters in superscript in the same row differ significantly (p<0.05).
